# Anxiety in Portugal: Associated Factors in Adult Population from 2011 to 2021

**DOI:** 10.3390/jcm14124100

**Published:** 2025-06-10

**Authors:** Ana Pedro Costa, Anabela Afonso, Irma da Silva Brito, Teresa Dionísio Mestre, Ana Matos Pires, Manuel José Lopes

**Affiliations:** 1 University of Évora, 7004-516 Évora, Portugal; tmestre82@gmail.com; 2 Comprehensive Health Research Centre, NOVA Medical School, Universidade NOVA de Lisboa, 1150-082 Lisboa, Portugal; ana.matos.pires@gmail.com (A.M.P.); mjl@uevora.pt (M.J.L.); 3 Psychiatry Service of Mental Health Department, Health Unit of Baixo Alentejo (ULSBA), 7801-849 Beja, Portugal; 4 Department of Mathematics, School of Science and Technology, University of Évora, 7000-671 Évora, Portugal; aafonso@uevora.pt; 5 Center for Research in Mathematics and Applications (CIMA), Institute for Advanced Studies and Research (IIFA), University of Évora, 7000-671 Évora, Portugal; 6 Nursing School of Coimbra, 3004-011 Coimbra, Portugal; irmabrito@esenfc.pt; 7 Health Sciences Research Unit: Nursing, 3046-851 Coimbra, Portugal; 8 Department of Health, Polytechnic Institute of Beja, 7800-295 Beja, Portugal; 9 São João de Deus School of Nursing, University of Évora, 7000-811 Évora, Portugal

**Keywords:** adults, anxiety, HADS, Portuguese population

## Abstract

**Background/Objectives**: Anxiety disorders are the most prevalent mental illnesses worldwide and in Portugal, often resulting in chronicity and disability. The objective of this study is to evaluate the sociodemographic and health-related factors associated with anxiety in the Portuguese adult population. **Methods**: This study included participants aged 18 to 65 years from the nationwide, population-based EpiDoC cohort, who were followed from 2011 to 2021 (n = 2927). Anxiety was assessed using the Hospital Anxiety and Depression Scale (HADS). A mixed logistic regression analysis was performed using a prospective analytical approach. Two strategies were used to adjust the mixed models: (i) model with only complete observations (n = 1950) and (ii) model with imputation of the category “No” in missing self-reported diseases (n = 2554). **Results**: The proportion of anxiety symptoms decreased from 2011–2013 to 2021 (12.5% vs. 8.5%). Experienced anxiety symptoms were positively associated (*OR* > 1, *p* < 0.05) with being female; having a high school, 2nd and 3rd cycle (6–9 years of studies), or primary/no education; being unemployed; seeking the first job; and not working or being temporarily unable to work. Additionally, anxiety symptoms were positively associated (*OR* > 1, *p* < 0.05) with smoking daily, lack of physical exercise, and medication use. Digestive diseases, multimorbidity, and region were also positively associated (*OR* > 1, *p* < 0.05) with anxiety symptoms. Moreover, age was negatively associated (*OR* < 1, *p* < 0.05) with experiencing anxiety symptoms. **Conclusions**: Some determinants are modifiable and preventable through economic, social, and health policies. Measures to promote healthy lifestyles, like physical exercise, reduce substance abuse, prevent chronic diseases, increase employability, and increase schooling and health literacy, are necessary to reduce the anxiety rate in Portugal.

## 1. Introduction

Anxiety involves the anticipation of a real or imagined future threat or danger and is often adaptive, playing a role in survival [1,2]. Many forms of anxiety are normal, such as performance anxiety, anxiety during life stress, or anxiety during life transitions [1,2]. However, anxiety may require clinical attention when it is disproportionate to the perceived threat, severe and persistent, or causes significant impairment in personal, family, social, educational, occupational, or other important areas of functioning [1,2]. Anxiety is characterized by somatic/physical symptoms such as shortness of breath, a “lump” in the throat, chills, tremors, and palpitations, and by psychological symptoms such as unease, discomfort, insecurity, and apprehension about the future [3]. It can also occur as a trait (a set of enduring and persistent personality characteristics), symptom (expression of a clinical condition), and state (when anxiety itself is the clinical condition) [4]. Anxiety disorders include clinical entities that share characteristics of excessive fear and anxiety and related behavioral changes; they differ in the types of objects or situations that induce fear, anxiety, or avoidance behavior, and in the associated cognition [5].

Anxiety disorders are the most common mental illness, with high prevalence, chronicity, comorbidity, and disability, marked by intense fear and distress and often accompanied by various physical symptoms [2,6,7]. According to the International Classification of Diseases 11th Revision (ICD-11), anxiety disorders include the following: generalized anxiety disorder, panic disorder, agoraphobia, specific phobia, social anxiety disorder, separation anxiety disorder, and selective mutism [8]. Anxiety disorders arise from multiple factors, including genetic influences (such as parental psychiatric conditions), psychobiological aspects (like temperament and personality), and environmental factors (such as parenting styles), along with childhood adversities and significant life events [7]. If not treated, chronic anxiety can lead to a heightened risk of developing cardiovascular disease, stroke, diabetes, arthritis, and lung disease compared to the general population [2,9]. Current treatments for anxiety generally include a combination of medication and psychotherapy. Pharmacological methods address the physiological symptoms associated with anxiety, while psychological approaches are needed to tackle the memories and triggers that cause anxiety [9].

Anxiety disorders are more common among women; young adults, at the individuals start of their career; individuals who are single; however, the protective factor is not being married, but being in a mutually supportive relationship; those with low educational attainment, because these individuals have less health literacy and therefore less healthy lifestyles; individuals who do not exercise, who smoke and consume alcohol; low income, but it was determined that anxiety disorders were higher in high-income, economically developed countries; people who are unemployed; people with chronic pathology, but in this case, the relationship may be bidirectional, since anxiety disorder is associated with a high risk of developing an illness and vice versa; and pain, a symptom of many diseases, can be associated with the onset of an anxiety disorder [2,9,10]. Proposed mechanisms for the higher occurrence of anxiety in individuals with somatic illnesses include unhealthy lifestyles, poor adherence to treatment, and dysregulation of psychobiological stress systems [2]. Additionally, inpatients experienced clinically significant anxiety symptoms; this underscores the commonality of anxiety in hospitalized populations and its potential impact on patient care [11]. The literature also states that anxiety disorders are associated with increased healthcare utilization across multiple care settings; this includes more frequent visits to primary care physicians, specialists, and emergency departments [12].

According to the World Health Organization (WHO) and Javaid et al. (2023) [9], in 2019, approximately 301 million people suffered from anxiety disorders worldwide, and Portugal had the highest prevalence, with 8671 cases per 100,000 people, followed by Brazil, Iran, and New Zealand [1,9]. WHO ranks anxiety disorders as the ninth leading cause of disability, which significantly impacts individuals’ daily functioning and quality of life and contributes to 3.3% of the global burden of disease, thereby placing a heavy burden on society [6]. These findings highlight the significant global impact of anxiety disorders [9], which are affected by population growth trends, socioeconomic status, the natural environment, and other contributing factors [6].

The risk factors for anxiety disorders are already well known in the general population. But the fact that Portugal has one of the highest prevalences of anxiety disorders is a phenomenon that needs to be analyzed. Considering the high prevalence of this disorder in Portugal and its impact, it is highly relevant to study its determinants and the associations between them. Thus, a research question arises as follows: What are the determinants of anxiety in the Portuguese adult population over the decade 2011 to 2021? Therefore, this study aimed to assess the factors associated with symptoms suggestive of anxiety in Portugal using a prospective analytical approach.

## 2. Materials and Methods

### 2.1. Study Design

This study used data from the Portuguese Epidemiology of Chronic Diseases (EpiDoC) Cohort, a nationwide prospective cohort that enrolled a nationally representative random sample of non-institutionalized Portuguese adults (≥18 years old). The cohort included four data collection periods, following a total of 10,661 participants over 10 years (2011–2021) [13].

### 2.2. Study Population

This study included participants who took part in both the EpiDoC1 and EpiDoC4 studies (hereafter referred to as t1 and t4), aged between 18 and 65 and between 2011 and 2013. Thus, the sample population consists only of non-institutionalized Portuguese adults aged between 18 and 65. To this end, and because the EpiDoC cohort has individuals aged >65 years, those aged ≥66 years were eliminated (n = 723). A total of 107 individuals were eliminated, as they only answered to the variable “date of interview”. No participants were removed from the sample due to incongruous answers. All the variables and questionnaires that did not fit within the scope of our study were eliminated, and the participant’s age at the time of the interview was corrected, since we detected that there were some differences between the age answered by the individual and the date of birth provided by them. Thus, this study included a sample of 2927 individuals.

### 2.3. Measurements and Instruments

#### 2.3.1. Outcome: Symptoms of Anxiety

In the two waves considered (EpiDoC1 and EpiDoC4), anxiety symptoms were measured using the Portuguese version of the Hospital Anxiety and Depression Scale (HADS) [14]. This self-report tool consists of 14 items, with seven assessing anxiety (HADS-A) and seven assessing depression (HADS-D). In this study, only the anxiety subscale (HADS-A) was used. Each item is rated on a four-point Likert scale, ranging from 0 (low) to 3 (high), with subscale scores ranging from 0 to 21. A cutoff score of 11 or higher indicates the presence of symptoms suggestive of anxiety (HADS-A ≥ 11) [15].

#### 2.3.2. Covariates

##### Sociodemographic Characteristics

Sociodemographic information was collected (age, gender, marital status, ethnicity, Nomenclature of Territorial Units for Statistics (NUTS), years of schooling) only at baseline (EpiDoC1). All socioeconomic variables (employment status and number of hours worked per week) were collected at both waves (EpiDoC1 and EpiDoC4, with a few exceptions).

##### Accessibility to Health Services

To assess healthcare resource consumption, the “number of medical appointments in the previous 12 months” and “hospitalization in the previous 12 months (yes/no)” were measured in both waves.

##### Health Status and Lifestyle Characteristics

In the EpiDoC1 study, individuals were asked whether they had been previously diagnosed with any chronic non-communicable disease, including high blood pressure, diabetes, dyslipidemia, chronic lung disease, heart disease, gastrointestinal disease, allergies, mental illness, thyroid or parathyroid disorders, hyperuricemia, urinary disease, or rheumatic disease. This information was updated during the EpiDoC4 study interview.

We define multimorbidity as having two or more self-reported chronic diseases, in line with Gupta et al. (2022) [16].

Medication use was measured in both waves by asking participants: “Are you currently taking any medication?” (yes/no).

Body mass index (BMI) was calculated based on self-reported height and weight and categorized as “underweight” (<18.5 kg/m^2^), “normal weight” (18.5–24.9 kg/m^2^), “overweight” (25–29.9 kg/m^2^), or “obese” (≥30 kg/m^2^).

Smoking habits were classified as “never”, “former”, “occasional”, or “daily”. Alcohol consumption was categorized as “Yes” or “No”.

Regular exercise was self-reported using the question: “Do you practice regular exercise or sports?” with response options of “Yes” or “No”.

### 2.4. Statistical Analysis

The data were processed and analyzed using the Statistical Package for the Social Sciences (SPSS), version 27 for Windows and version 28.0.0.0 (190) for Mac, and the R software, version 4.0.4 (R Foundation, Vienna, Austria).

Data analysis used the descriptive measures appropriate to each variable, i.e., measures of location and dispersion and absolute and relative frequencies (percentages). Kendall’s, Spearman’s, and Pearson’s correlation coefficients were used to measure the correlation between two variables, depending on the scale of the variables and the assumptions verified. Student’s *t*-test or ANOVA was used to compare the means of numeric variables. The chi-square test was used to test whether two categorical variables are related.

The consistency and reliability of the instrument used were assessed using Cronbach’s alpha coefficient. Alpha values between 0.70 and 0.95 are considered acceptable and appropriate [17]. For the instrument used (HADS-A), the Cronbach’s alpha coefficient was 0.78 at EpiDoC1 and 0.74 at EpiDoC4.

To evaluate the factors associated with anxiety, we combined some categories of a variable because they had few observations. This was the case with (i) ethnicity, where only the following two categories were defined: Caucasian and other; (ii) years of study, categorized as primary or lower (less than 6 years of study), 2nd and 3rd cycle (6–9 years of studies), high school (10–12 years of studies), and college/university level; (iii) professional status, where we defined the following seven categories: full-time active worker, part-time active worker, domestic worker, unemployed/seeking first job/not working, retired, student/working student; and (iv) the number of working hours per week (with the following answers: not an active worker, [1, 35) h, [35, 41) h, and [41, 100] h). One new variable was created to replace a previous one: “retired or unemployed due to illness”, with the following answers: “yes”, “no”, and “not applicable”. First, a mixed univariate logistic analysis was performed to examine the associations with each independent variable. Second, a mixed multiple logistic regression model was fitted with all variables that yielded a *p*-value of less than 0.25 in the mixed univariate analyses. Age, gender, and NUTS II were kept in the model by design. Plausible interactions among variables present in the multiple model were checked and were included if they were significant. AIC and BIC criteria were used in model selection. Residual analysis was performed to check the assumptions and the presence of outliers and influential observations. Multicollinearity was checked using the Generalized Variance-Inflation Factor (GVIF). The following two strategies were used to adjust the mixed models: (i) model without imputation: only complete observations in the variables of interest were used, resulting in a final sample of 1950 participants (approach 1), and (ii) model with imputation: the category “No” was imputed to all the individuals who answered “Do not know/No answer” to the self-reported diseases, resulting in a final sample of 2554 participants (approach 2). The area under the curve (AUC) for the model without imputation was 0.973, and for the model with imputation, it was 0.982.

## 3. Results

### 3.1. Sociodemographic, Socioeconomic, and Environmental Characteristics

At the baseline (t1), the average age was 45.18 ± 12.43 years, the majority were women (61.8%), approximately two-thirds were married (62.2%), and they were predominantly Caucasian (97.8%). Around one-third (31.5%) lived in the North, followed by 20.9% in the Center and only 2.7% in the South (i.e., Algarve). Just 2% had no more than two years of schooling, and the percentage of individuals with higher education is similar to those with high school, 2nd and 3rd cycle, or complete primary education (Table 1).

More than half of the participants were full-time workers (56.1% and 56.7% at t1 and t4, respectively, and 43.1% at both times), with an average of 40.52 ± 10.41 and 41.28 ± 10.44 h of work per week at t1 and t4, respectively. The percentage of individuals who retired due to illness is higher in t4 than in t1 (4.5% vs. 6.5%, *p* < 0.001), being 28.8% at both moments. Less than 1% were unemployed due to illness, and no one at both measurement times (Table 1).

### 3.2. Accessibility to Health Services

A large proportion of the individuals reported having appointments in the past 12 months, being significantly higher at t1 (87.1% vs. 77.1%, *p* < 0.001). In contrast, the proportion of individuals who were hospitalized/inpatient in the past 12 months was small and significantly lower in t1 (7.7% vs. 9.0%, *p* < 0.001). In the past 12 months, in both measurement moments, around two-thirds of the individuals attended appointments (68.7%), and 1.3% were hospitalized (Table 1).

### 3.3. Health Status and Lifestyle

The most common chronic diseases were high blood pressure (23.0% vs. 36.7%), cholesterol (25.6% vs. 38.6%), rheumatic disease (21.4% vs. 47.2%), mental illness (14.5% vs. 27.1%), and allergies (22.8% vs. 27.7%) (Table 1).

The percentage of individuals with multimorbidity increased from t1 to t4 (42.7% vs. 61.8%, *p* < 0.001), and 39.4% had multimorbidity at both t1 and t4. The chance of an individual having multimorbidity at t4 is 18.29 times higher for those who had multimorbidity at t1 compared to those who did not have multimorbidity at t1 (95% CI *OR*: 14.56–22.99) (Table 1).

The percentage of participants taking medication regularly increased between moments (48.8% vs. 63.2%, *p* < 0.001), and 43.1% took it at both moments (Table 1).

At t1, there was a predominance of individuals with a normal BMI (42.7%), while at t4, there was a higher percentage of participants with an overweight BMI (40.3%). The mean BMI was 26.39 ± 5.06 and 26.88 ± 4.70 at t1 and t4, respectively, and 29.8% of the individuals had normal weight at both t1 and t4 (Table 1).

It was found that there was a significant association between alcohol consumption, as well as tobacco consumption, with measurement moment (both *p* < 0.001). There were more individuals consuming alcohol daily at t4 (17.9% vs. 23.4%), and 12.9% at both measurement times. In contrast, there were fewer individuals daily consuming tobacco at t4 (18.4% vs. 15.7%), and 12.33% at both t1 and t4 (Table 1).

The percentage of individuals who do exercise differs between t1 and t4 (*p* < 0.001), observing a decrease from t1 to t4 (37.4% vs. 25.3%), and only 13.2% practiced physical exercise at both t1 and t4 (Table 1).

### 3.4. Anxiety Symptoms

The average score of the HADS instrument for anxiety (HADS-A) was 5.55 ± 3.95 pts and 4.81 ± 3.79 pts at t1 and t4, respectively. There is a statistically significant difference in the mean anxiety score between t1 and t4 (*t*(2846) = −9.655, *p* < 0.001), being 0.71 pts lower in t4. The percentage of individuals with anxiety scores greater than or equal to 11 on the HADS-A instrument was higher at t1 (12.5% vs. 8.5%, *p* < 0.001), and 4.5% had anxiety symptoms at both times. Individuals with anxiety in t1 have 11.15 more chances of having anxiety in t4 than individuals without anxiety at t1 (95% CI *OR*: 8.39–14.80). There is also a significant positive and moderately weak linear relation between the HADS-A scores recorded at t1 and t4 (*r* = 0.484, *p* < 0.001), i.e., there is a tendency for individuals with high levels of anxiety at t1 to also have high levels of anxiety at t4 (Table 1).

### 3.5. Factors Associated with Anxiety Symptoms

The results of the univariate mixed logistic models, based on the complete case sample (*n* = 1950, 84.3% had HADS-A score < 11 at t1 and t4), approach 1, and the complete sample after imputing values in the self-reported diseases (*n* = 2554, 84.0% had HADS-A score < 11 at t1 and t4), approach 2, were generally in agreement both in the associations identified and in the value of the effect. Both approaches revealed that gender, level of education, region of residence, age, employment status, number of hours worked per week, alcohol and tobacco consumption, physical exercise, taking medication, having multimorbidity, diseases of the digestive system, allergies, mental, urinary, and rheumatic diseases, history of medical appointments in the past 12 months, and history of hospitalizations in the past 12 months are associated with the presence of anxious symptoms. In contrast, both approaches revealed that ethnicity, body mass index, and diseases like high blood pressure, diabetes, heart disease, and hyperuricemia are not associated with anxiety (all *p* > 0.05). Differences were found between the approaches for the variables of being retired or unemployed due to illness, living in Lisbon, and having diseases like oncological, neurological, pulmonary, and high cholesterol levels. However, the magnitude of the effect was similar in both approaches. All these variables were only statistically significant in the models that considered data with imputation (all *p* < 0.05, Table 2).

After adjusting for the presence of other variables (multiple models; Table 3) and retaining just the significant variables, in both approaches the marital status, being retired due to illness/unemployed due to illness, the diseases of high cholesterol level, pulmonary, neurological, allergic, oncological, and rheumatic, and the history of medical appointments are no longer significant to explain the presence of the anxiety symptoms (all *p* > 0.05). The effect of gender decreased (*OR*: 7.53–8.42, adj. *OR*: 5.09–5.67), as well as the effect of none/primary level of education (*OR*: 6.03–5.63, adj. *OR*: 4.5–4.7) and taking medication (*OR*: 3.47–2.91, adj. *OR*: 1.9), suggesting that some of their effect is explained by other covariates. Conversely, there was an increase in the effect of unemployed/seeking first job/not working (*OR*: 0.93–4.1, adj. *OR*:1.96–8.07).

There are some differences between the multiple models. Model without imputation (model 1) maintained as significant factors for a person to have anxiety symptoms, have mental disease, and have a history of hospitalization. The model with imputation (model 2) kept as significant factors working more than 40 h a week, having hypertension disease, having urinary disease, and not consuming alcohol.

Also, the included interaction terms are not the same in both models. In the model without imputation (model 1), there is a significant interaction between time and digestive disease. Individuals with and without digestive disease have lower chances of having anxiety at t4 than at t1 (both adj. *OR* < 1). At t1, individuals with digestive disease have a higher possibility of having anxiety than individuals without this disease. In the model with imputation (model 2), there is a significant interaction between time and multimorbidity and between time and region. In t1, multimorbidity is associated with more chances of having anxiety (adj. *OR* = 3.52), while in t4, it is not significant. In almost every region, the chances of individuals with multimorbidity having anxiety are lower at t4 than at t1. Also, in the North and Center, individuals without multimorbidity have lower chances of having anxiety at t4 than at t1.

With both multiple models, we can conclude that the chances of an individual having anxiety symptoms are (i) 5 times higher for females, (ii) 2 times higher for those who have high school or 2nd and 3rd cycle, and 5 times higher for the ones with primary/none education when compared with college/university level, (iii) decrease with age, (iv) are higher for the unemployed and for individuals seeking their first job or not working or with a temporary incapacity for work when compared to full-time active workers, (v) are 2 times more to those who daily consume tobacco, (vi) are around 1.5 times higher for the ones that do not do physical exercise, and (vii) are 2 times higher for those who take medication.

## 4. Discussion

This study revealed a percentage of anxiety symptoms of 12.5% and 8.5% of the participants (Portuguese people with ages between 18 and 65 years of age) at t1 (2011–2013) and t4 (2021), respectively. Without adjusting for the presence of other variables, the estimated chances of having anxiety symptoms at t4 are around 50% less than at t1.

The prevalence of anxiety among the Portuguese population has increased in recent years, especially during and after the COVID-19 pandemic. Santos et al. (2022) report that around 9% of the Portuguese population were diagnosed with an anxiety disorder in 2022 versus 8% in 2019 [18]. Javaid et al. (2023) [9], who examined the global and regional burden of anxiety disorders through analyzing epidemiological data from the Global Burden of Disease dataset, found a prevalence in Portugal of 8.67% in 2019 [9]. This is the highest of the 204 countries and regions included; Spain presented a prevalence of 5.13% and France of 6.56% [9]. However, in the present study, we observed a decreased proportion of the anxiety symptoms between 2011 and 2013 and 2021 by monitoring the ageing of the individuals in the sample studied. This difference could be explained by the fact that anxiety decreases with age in this study, which is in line with what is reported in the literature as follows: Anxiety generally decreases with advancing age, with young adults showing higher levels of anxiety than older people [19]. Also, self-report questionnaires can underestimate prevalence, depending on the context and the participants’ interpretation of symptoms [20]. The HADS-A is useful for recognizing anxiety symptoms, but it is not ideal for assessing the prevalence of anxiety in the general population [21]. Although it is reliable and has good psychometric properties, it has been developed for screening, which limits its application for estimating general prevalence; it is preferable to use more specific and broader instruments, such as standardized diagnostic interviews [21].

Our results obtained from both regression models are consistent with those found in other studies. Women have higher levels of anxiety than men due to biological and sociocultural factors [22]. Adults having low educational levels tend to have higher levels of anxiety associated with economic concerns and social expectations [23]. Unemployed people are more likely to report higher levels of anxiety because employment plays a pivotal role in not only meeting fundamental survival requirements through financial security but also in fulfilling other fundamental human needs [24]. The job search process generates anxiety due to the unpredictability of results and the pressure for professional success, especially among young university students who are starting their careers [25]. Daily smokers have a higher prevalence of anxiety, with a positive association between smoking and mental illness, with smoking rates increasing with the severity of the illness [26]. Lack of regular physical activity is associated with an increased level of anxiety, while exercise has positive effects on mental health [27]. Recent studies have shown a link between cholesterol disorders and mental health conditions, especially anxiety; additionally, research suggests that cholesterol-lowering drugs, like statins, may influence mental health, and other chronic diseases, such as kidney disease, coronary heart disease, and asthma, have also been associated with mental health issues [28]. By delineating the profile of the Portuguese population, it becomes possible to design and implement targeted early intervention strategies. These findings highlight the importance of preventive policies focused on (i) academic progression and achievement; (ii) inclusive labor market strategies aimed at reducing unemployment and increasing job opportunities; (iii) initiatives to reduce substance use, particularly tobacco consumption; (iv) structured physical activity programs incorporating regular exercise; and (v) effective public health strategies for the prevention and control of chronic diseases.

The results about alcohol intake were not congruent in both of our models (not associated vs. associated). While this outcome may initially appear concerning, it is crucial to remember that this study evaluated how often consumption occurred rather than the specific types or amounts consumed. However, the literature posits that the relationship between anxiety and alcohol consumption is bidirectional, where people with anxiety often resort to alcohol as a form of self-medication, which, despite initially relieving symptoms, can worsen them and increase the risk of dependence; in addition, chronic alcohol consumption increases vulnerability to the development of anxiety disorders [29]. Nevertheless, as with tobacco, as mentioned above, it is important to promote intervention strategies to minimize alcohol consumption and the risks associated with it.

There is a well-established link between anxiety and digestive disorders, with research showing that psychological stress can affect gut health and worsen gastrointestinal diseases. This is often attributed to the gut–brain axis, a communication system between the central nervous system and the enteric nervous system, which anxiety can disrupt, leading to digestive issues [30]. We detected a significant interaction between time and digestive disease (model 1). At t1, individuals with digestive disease have a higher possibility of having anxiety than individuals without this disease. It might be worth considering whether it makes sense to screen all individuals with anxiety symptoms for digestive diseases in a clinical context, and the other way around, i.e., to assess the presence of anxiety symptoms in individuals with known digestive diseases. For a more accurate assessment, more studies are needed that address these issues.

Our results revealed that multimorbidity is associated with higher rates of anxiety at t1, but at t4, this was not significant (model 2). The study conducted by Vancampfort et al. (2017) indicates that a growing number of chronic physical conditions (angina, arthritis, asthma, chronic back pain, diabetes, edentulism, hearing problems, tuberculosis, and visual impairment) are linked to an increased likelihood of anxiety and that health care providers should consider the presence of anxiety symptoms, especially in individuals with physical multimorbidity [31]. Huang et al. (2023) showed that cholesterol disease, kidney disease, coronary heart disease, and asthma are significantly associated with mental health concerns [28]. The odd result at t4, which should also be significant since individuals have aged and are more likely to have accumulated more diseases and therefore have more multimorbidity, can be explained by the fact that anxiety decreases with age in this study, which is consistent with the literature as reported above.

Several studies have used and published papers using the EpiDoC database; one of them also studied anxiety, but in older individuals [32]. The study by Sousa et al. (2017) [32], which used data from EpiDoC1 (2011–2013) and EpiDoC2 (2013–2015) to assess anxiety in elderly people (≥65 years) found an estimated prevalence of anxious symptoms of 9.6%. In addition, they concluded that women, low levels of education, allergies, and rheumatic diseases were significantly and independently associated with the presence of anxiety symptoms [32]. Even though we were studying another age group, and therefore the individuals included in our study were not the same, we found a similar proportion, and our approaches also found identical conclusions.

By using the EpiDoC cohort, this study has several strengths and limitations. Among the strengths is the broad representativeness, since EpiDoC covers a national sample representative of Portugal, although the population studied at the end of this study is no longer so, but it still allows a detailed demographic analysis of the Portuguese population, especially in relation to chronic diseases. Furthermore, being a longitudinal study makes it easier to monitor the progression of health conditions over time. Another strength is the holistic approach, which combines not only demographic data but also socioeconomic and environmental characteristics and accessibility to health services, as well as results from scales, making it possible to measure medical conditions and self-report many chronic diseases. Regarding limitations, one of them is the participant attrition over time, which is common in longitudinal studies and impacts the representativeness of the data. In addition, part of the data is self-reported, especially on health and lifestyle issues, which can introduce some bias of social acceptability. The data are community-based and so imbalanced, with most of the participants having a HADS-A score < 11. This can lead to biased parameter estimates and affect model performance. While data balancing is a widely accepted practice, undersampling would greatly reduce the sample size, and oversampling would generate synthetic data that may not fully reflect the real-world distribution. Given these limitations, generalizing the results to the broader population should be performed with caution.

## 5. Conclusions

This study aimed to evaluate the sociodemographic and health-related factors associated with anxiety in the Portuguese adult population. The most significant results of this study highlight the following: (i) the proportion of anxiety symptoms in our sampling, when compared with the literature, is similar: (ii) the likelihood of an individual experiencing anxiety symptoms is higher among females, those with high school, 2nd and 3rd cycle, or primary/no education, unemployed individuals seeking their first job, those not working or temporarily incapacitated to work, and is also higher among those who smoke daily, do not engage in physical exercise, or take medication, while the likelihood decreases with age; and (iii) the only variables that interacted with time were digestive diseases, multimorbidity, and region.

Several of these determinants are modifiable and, as such, can be addressed through the implementation of targeted economic, social, health, and well-being policies. It is essential to adopt comprehensive measures aimed at promoting physical activity, curbing substance abuse, preventing the onset of chronic diseases, enhancing employability, and improving both education and health literacy. By advancing these initiatives, it becomes possible to mitigate key risk factors that contribute to anxiety disorders. In Portugal, such interventions are particularly crucial, as they can play a significant role in reducing the national anxiety rate and fostering a healthier, more sustained sense of well-being in the population.

Further studies will be conducted to explore the association between anxiety symptoms, quality of life, and physical functioning in this population.

## Figures and Tables

**Table 1:** Sociodemographic, socioeconomic, environmental characteristics, accessibility to health, health status, and lifestyle characteristics of the sample across the two waves (*N* = 2927).

		T1		T4			
		*N* (%)	NA, *N* (%)	*N* (%)	NA, *N* (%)	*X*2 (d.f.)	*p*
Sociodemographic							
Age * (in years)	M (SD)	45.19 ± 12.46	0 (0.0)				
	Median	46					
	Range	18–65					
Gender *	Female	1809 (61.8)	0 (0.0)				
	Male	1118 (38.2)					
Marital status *	Married	1821 (62.2)	1 (0.0)				
	Single	612 (20.9)					
	Divorced	251 (8.6)					
	Widowed	124 (4.2)					
	Nonmarital partnership	118 (4.0)					
Ethnicity *	Caucasian	2864 (97.9)	3 (0.1)				
	Black	46 (1.6)					
	Gypsy	2 (0.1)					
	Asian	1 (0.0)					
	Other	11 (0.4)					
Region *	North	923 (31.5)	0 (0.0)				
	Lisbon	680 (23.2)					
	Center	562 (19.2)					
	Azores	295 (10.1)					
	Madeira	226 (7.7)					
	Alentejo	161 (5.5)					
	Algarve	80 (2.7)					
Education level *	College/university	760 (26.0)	4 (0.1)				
	High school	709 (24.2)					
	2nd and 3rd cycle	645 (22.0)					
	Primary	751 (25.7)					
	None	58 (2.0)					
Employment status	Full-time active worker	1641 (56.1)	20 (0.7)	1659 (56.7)	27 (0.9)		
	Active part-time worker	116 (4.0)		98 (3.3)			
	Domestic worker	205 (7.0)		163 (5.6)			
	Unemployed	327 (11.2)		158 (5.4)			
	Retired	362 (12.4)		698 (23.8)			
	Student	152 (5.2)		13 (0.4)			
	Temporary incapacity for work	54 (1.8)		111 (3.8)			
	Not working but living on income *	23 (0.8)					
	Looking for first job *	12 (0.4)					
	Worker-Student *	15 (0.5)					
No. of working hours/week	M (SD)	40.52 ± 10.41	27 (1.5)	41.28 ± 10.44	20 (1.1)		
	Median	40		40			
	Range	1–90		4–93			
Retirement due to illness	No	229 (7.8)	1 (0.0)	496 (16.9)	123 (4.2)	194.19 (1)	<0.001
	Yes	132 (4.5)		190 (6.5)			
Unemployment due to illness	No	296 (10.1)	2 (0.1)	136 (4.7)	8 (0.3)		
	Yes	29 (1.0)		14 (0.5)			
Accessibility to health services							
Medical appointments	No	379 (12.9)	0 (0.0)	623 (21.3)	48 (1.6)	41.80 (1)	<0.001
	Yes	2548 (87.1)		2256 (78.4)			
Hospitalized/ inpatient	No	2703 (92.3)	0 (0.0)	2644 (90.3)	20 (0.7)	17.72 (1)	<0.001
	Yes	224 (7.7)		263 (9.0)			
Health status and lifestyle							
Hypertension	No	2238 (76.5)	16 (0.5)	1473 (50.3)	381 (13.0)		
	Yes	673 (23.0)		1073 (36.7)			
Diabetes mellitus	No	2714 (92.7)	17 (0.6)	2066 (70.6)	550 (18.8)		
	Yes	196 (6.7)		311 (10.6)			
Cholesterol	No	2149 (73.4)	29 (1.0)	1297 (44.3)	499 (17.0)		
	Yes	749 (25.6)		1131 (38.6)			
Hyperuricemia	No	2767 (94.5)	0 (0.0)	2151 (73.5)	0 (0.0)		
	Yes	135 (4.6)		192 (6.6)			
Pulmonary disease	No	2789 (95.3)	13 (0.4)	2136 (73.0)	574 (19.6)		
	Yes	125 (4.3)		217 (7.4)			
Cardiac disease	No	2716 (92.8)	22 (0.8)	1999 (68.3)	550 (18.8)		
	Yes	189 (6.5)		378 (12.9)			
Digestive disease	No	2495 (85.1)	19 (0.6)	1776 (60.7)	551 (18.8)		
	Yes	413 (14.1)		600 (20.5)			
Neurological disease	No	2849 (97.3)	11 (0.4)	2122 (72.5)	541 (18.5)		
	Yes	67 (2.3)		264 (9.0)			
Oncological disease	No	2826 (96.5)	14 (0.5)	2156 (73.7)	554 (18.9)		
	Yes	87 (3.0)		217 (7.4)			
Urinary disease	No	2710 (92.6)	17 (0.6)	2035 (69.5)	561 (19.2)		
	Yes	200 (6.8)		331 (11.3)			
Rheumatic disease	No	2226 (76.1)	74 (2.5)	1530 (52.3)	14 (0.5)		
	Yes	627 (21.4)		1383 (47.2)			
Mental disorder	No	2493 (85.2)	9 (0.3)	1629 (55.7)	506 (17.3)		
	Yes	425 (14.5)		792 (27.1)			
Allergies	No	2236 (76.4)	23 (0.8)	1577 (53.9)	539 (18.4)		
	Yes	668 (22.8)		811 (27.7)			
Multimorbidity	No	1676 (57.3)	0 (0.0)	1118 (38.2)	0 (0.0)	850.94 (1)	<0.001
	Yes	1251 (42.7)		1809 (61.8)			
Take medication	No	1491 (50.9)	7 (0.2)	1067 (36.5)	11 (0.4)	777.51 (1)	<0.001
	Yes	1429 (48.9)		1849 (63.2)			
Body mass index (BMI)	M (SD)	26.39 ± 5.06	57 (1.9)	26.88 ± 4.7	89 (3.0)		
	Median	26		26			
	Range	15–68		15–60			
	Underweight (<18.5)	44 (1.5)		29 (1.0)			
	Normal (18.5–24.9)	1190 (42.7)		1027 (36.8)			
	Overweight (25–29.9)	1107 (36.5)		1124 (40.3)			
	Obesity (≥30)	537 (19.3)		608 (21.8)			
Alcohol consumption	Never	1136 (38.9)	3 (0.1)	1102 (37.6)	29 (1.0)	1338.77 (2)	<0.001
	Occasionally	1265 (43.2)		1111 (38.0)			
	Daily	523 (17.9)		685 (23.4)			
Tobacco consumption	Never	1731 (59.1)	1 (0.0)	1745 (59.6)	26 (0.9)	3109.12 (3)	<0.001
	In the past	571 (19.5)		650 (22.2)			
	Occasionally	84 (2.9)		46 (1.6)			
	Daily	540 (18.4)		460 (15.7)			
Physical exercise	No	1831 (62.6)	1 (0.0)	2155 (73.6)	31 (1.1)	90.85 (1)	<0.001
	Yes	1095 (37.4)		741 (25.3)			
Anxiety							
HADS-A Score	M (SD)	5.55 ± 5	0 (0)	4.81 ± 4	80 (2.7)		
	Median	3		2			
	Range	0–21		0–20			
	HADS-A < 11	2561 (87.5)		2599 (88.8)		377.46 (1)	<0.001
	HADS-A ≥ 11	366 (12.5)		248 (8.5)			

* Not included in the response options for at t4. Note: *N* = absolute frequencies, % = relative frequency, NA = missing value due to ‘do not know’ or non-response, M = mean, SD = standard deviation, *X*2 = chi-square test, d.f. = degrees of freedom, *p* = *p*-value.

**Table 2:** Univariate logistic mixed effects regression models for anxiety symptoms: approach 1: using complete cases (*N* = 1950), and approach 2: using data with imputed values in self-reported chronic non-communicable diseases (*N* = 2554).

	Approach 1			Approach 2		
Covariates	*OR*	(95% CI)	*p*	*OR*	(95% CI)	*p*
Age (scaled)	**1.25**	(1.04, 1.52)	0.020	**1.35**	(1.15, 1.60)	<0.001
Gender (ref. = male)	**8.42**	(5.30, 14.10)	<0.001	**7.53**	(5.10, 11.60)	<0.001
Marital status (ref. = single)						
Married	**2.12**	(1.28, 3.53)	0.004	**2.57**	(1.63, 4.06)	<0.001
Divorced	**2.46**	(1.16, 5.22)	0.020	**3.49**	(1.81, 6.74)	<0.001
Widowed	**7.39**	(2.81, 19.41)	<0.001	**8.32**	(3.65, 18.98)	<0.001
Nonmarital partnership	0.98	(0.34, 2.87)	0.973	1.48	(0.60, 3.65)	0.396
Ethnicity (ref. = Caucasian)	0.56	(0.15, 2.15)	0.401	0.62	(0.19, 2.08)	0.442
Region (ref. = North)						
Center	**0.55**	(0.32, 0.96)	0.036	**0.52**	(0.32, 0.84)	0.008
Lisbon	0.68	(0.41, 1.13)	0.139	**0.59**	(0.38, 0.91)	0.016
Alentejo	**0.37**	(0.15, 0.94)	0.036	**0.41**	(0.18, 0.92)	0.029
Algarve	0.29	(0.07, 1.20)	0.088	0.56	(0.19, 1.61)	0.283
Azores	1.42	(0.76, 2.65)	0.273	1.18	(0.68, 2.05)	0.552
Madeira	0.74	(0.35, 1.56)	0.432	0.75	(0.39, 1.44)	0.394
Level of education (ref. = college/university)						
High school	1.62	(0.93, 2.87)	0.088	1.63	(1.00, 2.66)	0.050
2nd and 3rd cycle	**2.79**	(1.61, 4.94)	<0.001	**2.65**	(1.64, 4.36)	<0.001
None/primary	**6.03**	(3.58, 10.60)	<0.001	**5.63**	(3.58, 9.16)	<0.001
Employment status (ref. = full-time active worker)						
Part-time worker	4.39	(0.67, 3.05)	0.360	1.54	(0.79, 3.01)	0.201
Domestic worker	**4.11**	(2.48, 8.33)	<0.001	**4.99**	(2.98, 8.37)	<0.001
Unemployed/seeking first job/not working	**0.83**	(2.59, 6.99)	<0.001	**4.10**	(2.69, 6.23)	<0.001
Retired	0.26	(0.52, 1.41)	0.545	0.97	(0.64, 1.46)	0.884
Student/working student	6.70	(0.10, 1.21)	0.098	**0.24**	(0.07, 0.81)	0.021
Temporary incapacity for work	**4.39**	(3.39, 14.00)	<0.001	**5.73**	(3.03, 10.81)	<0.001
Number of working hours/week						
(ref. = not an active worker)						
[1, 35)	0.57	(0.28, 1.11)	0.108	0.57	(0.30, 1.03)	0.071
[35, 41)	**0.46**	(0.32, 0.67)	<0.001	**0.45**	(0.33, 0.62)	<0.001
[41, 100]	**0.51**	(0.31, 0.82)	0.006	**0.57**	(0.38, 0.86)	0.007
Retired due to illness/unemployed due to illness (ref. = no)						
Not applicable	0.93	(0.62, 1.40)	0.736	0.93	(0.65, 1.31)	0.664
Yes	2.05	(0.98, 4.29)	0.056	**2.14**	(1.16, 3.97)	0.015
Medical appointments (ref. = no)	**2.04**	(1.30, 3.27)	0.002	**2.06**	(1.40, 3.09)	<0.001
Hospitalized/inpatient (ref. = no)	**2.37**	(1.41, 3.94)	0.001	**2.91**	(2.18, 3.93)	<0.001
Hypertension (ref. = no)	1.19	(0.81, 1.75)	0.369	1.26	(0.91, 1.74)	0.161
Diabetes mellitus (ref. = no)	1.30	(0.71, 2.38)	0.387	1.50	(0.89, 2.50)	0.117
Cholesterol (ref. = no)	1.31	(0.91, 1.87)	0.136	**1.49**	(1.10, 2.02)	0.010
Hyperuricemia (ref. = no)	1.50	(0.73, 3.02)	0.258	1.61	(0.85, 2.98)	0.135
Pulmonary disease (ref. = no)	1.84	(0.92, 3.61)	0.078	1.80	(1.00, 3.17)	0.044
Cardiac disease (ref. = no)	1.56	(0.90, 2.64)	0.104	1.56	(0.98, 2.45)	0.057
Digestive disease (ref. = no)	**2.80**	(1.87, 4.23)	<0.001	**3.04**	(2.13, 4.37)	<0.001
Neurological disease (ref. = no)	1.69	(0.93, 2.99)	0.077	**1.86**	(1.11, 3.08)	0.017
Oncological disease (ref. = no)	1.92	(0.95, 3.77)	0.062	**1.93**	(1.06, 3.46)	0.029
Urinary Disease (ref. = no)	**2.33**	(1.34, 4.05)	0.002	**2.51**	(1.57, 4.03)	<0.001
Rheumatic disease (ref. = no)	**2.09**	(1.52, 2.89)	<0.001	**2.28**	(1.72, 3.01)	<0.001
Mental disorder (ref. = no)	**6.97**	(5.08, 9.68)	<0.001	**6.53**	(4.95, 8.69)	<0.001
Allergies (ref. = no)	**1.67**	(1.14, 2.43)	0.008	**1.57**	(1.13, 2.18)	0.007
Multimorbidity (ref. = no)	**2.78**	(2.02, 3.85)	<0.001	**3.25**	(2.47, 4.32)	<0.001
Take medication (ref. = no)	**3.47**	(2.46, 4.96)	<0.001	**2.91**	(2.18, 3.93)	<0.001
BMI (ref. = underweight)						
Normal	1.67	(0.33, 8.38)	0.535	1.42	(0.35, 5.73)	0.622
Overweight	1.62	(0.32, 8.19)	0.559	1.38	(0.34, 5.88)	0.654
Obesity	2.10	(0.41, 10.81)	0.377	1.71	(0.41, 7.06)	0.458
Alcohol consumption (ref. = never)						
Occasionally	**0.40**	(0.28, 0.56)	<0.001	**0.44**	(0.33, 0.60)	<0.001
Daily	**0.28**	(0.17, 0.45)	<0.001	**0.29**	(0.19, 0.43)	<0.001
Tobacco consumption (ref. = never)						
In the past	**0.55**	(0.35, 0.89)	0.015	**0.57**	(0.38, 0.85)	0.006
Occasionally	0.54	(0.16, 1.90)	0.340	0.46	(0.15, 1.38)	0.165
Daily	1.31	(0.83, 2.06)	0.248	1.18	(0.79, 1.76)	0.407
Physical exercise (ref. = yes)	**1.65**	(1.17, 2.35)	0.005	**1.60**	(1.19, 2.17)	0.002
Time (ref. = t1)	**0.57**	(0.43, 0.75)	<0.001	**0.53**	(0.42, 0.68)	<0.001

Note: *OR* = odds ratio, 95% CI = *OR* 95% confidence interval, *p* = Wald test *p*-value. When bold: (*OR* > 1, *p* < 0.05) or (*OR* < 1, *p* < 0.05).

**Table 3:** Multiple logistic mixed effects regression models for anxiety symptoms: model 1 without imputation (*N* = 1950), model 2 with imputed values in auto-reported diseases (*N* = 2554).

	Model 1				Model 2	
Covariates	*Adj. OR*	(95% CI)	*p*	*Adj. OR*	(95% CI)	*p*
Age (scaled)	**0.78**	(0.61, 0.99)	0.044	0.78	(0.62, 0.98)	0.031
Gender (ref. = male)	**5.09**	(3.17, 8.19)	<0.001	**5.67**	(3.62, 8.89)	<0.001
Region (ref. = north)						
Center	0.66	(0.39, 1.09)	0.106	**0.37**	(0.21, 0.66)	0.001
Lisbon	0.88	(0.55, 1.41)	0.593	0.70	(0.42, 1.18)	0.183
Alentejo	0.48	(0.20, 1.13)	0.093	**0.23**	(0.08, 0.63)	0.004
Algarve	0.53	(0.15, 1.93)	0.338	0.95	(0.30, 3.06)	0.932
Azores	0.95	(0.54, 1.68)	0.852	0.59	(0.30, 1.14)	0.116
Madeira	0.69	(0.34, 1.37)	0.287	0.48	(0.22, 1.05)	0.067
Level of education (ref. = college/university)						
High school	**1.81**	(1.07, 3.08)	0.027	**1.73**	(1.06, 2.81)	0.028
2nd and 3rd cycle	**2.41**	(1.40, 4.16)	0.002	**2.29**	(1.38, 3.79)	0.001
None/primary	**4.50**	(2.52, 8.04)	<0.001	**4.70**	(2.75, 8.05)	<0.001
Employment status (ref. = full-time active worker)						
Part-time worker	0.74	(0.35, 1.58)	0.443	1.35	(0.57, 3.19)	0.500
Domestic worker	1.25	(0.67, 2.33)	0.474	5.51	(0.90, 33.61)	0.064
Unemployed/seeking first job/not working	**1.96**	(1.21, 3.17)	0.007	**8.07**	(1.34, 48.42)	0.022
Retired	0.59	(0.33, 1.05)	0.072	2.95	(0.49, 17.83)	0.239
Student/working student	0.43	(0.12, 1.51)	0.188	0.91	(0.13, 6.25)	0.921
Temporary incapacity for work	**3.89**	(1.95, 7.78)	<0.001	**16.47**	(2.57, 105.58)	0.003
Number of working hours/week						
(ref. = not an active worker)						
[1, 35)				2.52	(0.41, 15.36)	0.316
[35, 41)				3.40	(0.59, 19.62)	0.171
[41, 100]				**6.07**	(1.02, 36.21)	0.048
Hospitalized/inpatient (ref. = no)	**1.70**	(1.03, 2.81)	0.039			
Hypertension (ref. = no)				**0.57**	(0.39, 0.84)	0.004
Digestive disease (ref. = no)				**1.80**	(1.23, 2.64)	0.003
Urinary disease (ref. = no)				**1.78**	(1.09, 2.9)	0.020
Mental disorder (ref. = no)	**5.31**	(3.67, 7.68)	<0.001			
Take medication (ref. = no)	**1.90**	(1.30, 2.79)	0.001	**1.90**	(1.34, 2.7)	<0.001
Alcohol consumption (ref. = never)						
Occasionally				**0.68**	(0.49, 0.95)	0.022
Daily				**0.55**	(0.35, 0.87)	0.011
Tobacco consumption (ref. = never)						
In the past	1.17	(0.73, 1.86)	0.517	1.22	(0.80, 1.87)	0.353
Occasionally	0.70	(0.19, 2.63)	0.597	0.82	(0.24, 2.76)	0.749
Daily	**2.20**	(1.40, 3.46)	0.001	**2.06**	(1.35, 3.14)	0.001
Physical exercise (ref. = yes)	**1.49**	(1.05, 2.12)	0.027	**1.51**	(1.10, 2.08)	0.012
Digestive disease (no): Time (ref. = t1)	**0.47**	(0.33, 0.67)	<0.001			
Digestive disease (yes): Time (ref. = t1)	**0.18**	(0.10, 0.32)	<0.001			
Time (t1): Digestive disease (ref. = no)	**2.73**	(1.64, 4.55)	<0.001			
Time (t4): Digestive disease (ref. = no)	1.04	(0.61, 1.76)	0.890			
Any region						
Time (t1): Multimorbidity (ref. = no)				**3.52**	(2.30, 5.38)	<0.001
Time (t4): Multimorbidity (ref. = no)				1.59	(0.98, 2.59)	0.060
No multimorbidity						
Region (North): Time (ref. = t1)				**0.49**	(0.28, 0.84)	0.010
Region (Center): Time (ref. = t1)				1.04	(0.52, 2.11)	0.900
Region (Lisbon): Time (ref. = t1)				0.54	(0.28, 1.01)	0.050
Region (Alentejo): Time (ref. = t1)				1.75	(0.52, 5.89)	0.370
Region (Algarve): Time (ref. = t1)				**0.13**	(0.02, 0.84)	0.030
Region (Azores): Time (ref. = t1)				0.99	(0.45, 2.17)	0.980
Region (Madeira): Time (ref. = t1)				1.12	(0.43, 2.9)	0.820
Multimorbidity						
Region (North): Time (ref. = t1)				**0.22**	(0.14, 0.36)	<0.001
Region (Center): Time (ref. = t1)				**0.47**	(0.25, 0.89)	0.020
Region (Lisbon): Time (ref. = t1)				**0.24**	(0.14, 0.43)	<0.001
Region (Alentejo): Time (ref. = t1)				0.79	(0.25, 2.52)	0.690
Region (Algarve): Time (ref. = t1)				**0.06**	(0.01, 0.37)	<0.001
Region (Azores): Time (ref. = t1)				**0.45**	(0.22, 0.92)	0.030
Region (Madeira): Time (ref. = t1)				0.51	(0.20, 1.26)	0.140

Note: *Adj. OR* = adjusted odds ratio, 95% CI = *OR* 95% confidence interval, *p* = Wald test *p*-value. When bold: (*OR* > 1, *p* < 0.05) or (*OR* < 1, *p* < 0.05).

## Data Availability

To answer the proposed research question, we used data from the Portuguese Epidemiology of Chronic Diseases (EpiDoC) cohort. EpiDoC is a prospective and longitudinal cohort study with a sequence of random samples, representative of the non-institutionalized Portuguese adult population (≥18 years of age), including mainland Portugal and the islands (Azores and Madeira) [13,31,32].
